# MRI-Derived Dural Sac and Lumbar Vertebrae 3D Volumetry Has Potential for Detection of Marfan Syndrome

**DOI:** 10.3390/diagnostics14121301

**Published:** 2024-06-19

**Authors:** Omar Naas, Tobias Norajitra, Christian Lückerath, Matthias A. Fink, Klaus Maier-Hein, Hans-Ulrich Kauczor, Fabian Rengier

**Affiliations:** 1 Clinic for Diagnostic and Interventional Radiology, Heidelberg University Hospital, Im Neuenheimer Feld 420, 69120 Heidelberg, Germany; 2 Division of Medical Image Computing, German Cancer Research Center (DKFZ), Im Neuenheimer Feld 280, 69120 Heidelberg, Germany

**Keywords:** machine learning, Marfan syndrome, magnetic resonance imaging, dural ectasia, volumetry

## Abstract

Purpose: To assess the feasibility and diagnostic accuracy of MRI-derived 3D volumetry of lower lumbar vertebrae and dural sac segments using shape-based machine learning for the detection of Marfan syndrome (MFS) compared with dural sac diameter ratios (the current clinical standard). Materials and methods: The final study sample was 144 patients being evaluated for MFS from 01/2012 to 12/2016, of whom 81 were non-MFS patients (46 [67%] female, 36 ± 16 years) and 63 were MFS patients (36 [57%] female, 35 ± 11 years) according to the 2010 Revised Ghent Nosology. All patients underwent 1.5T MRI with isotropic 1 × 1 × 1 mm^3^ 3D T2-weighted acquisition of the lumbosacral spine. Segmentation and quantification of vertebral bodies L3-L5 and dural sac segments L3-S1 were performed using a shape-based machine learning algorithm. For comparison with the current clinical standard, anteroposterior diameters of vertebral bodies and dural sac were measured. Ratios between dural sac volume/diameter at the respective level and vertebral body volume/diameter were calculated. Results: Three-dimensional volumetry revealed larger dural sac volumes (*p* < 0.001) and volume ratios (*p* < 0.001) at L3-S1 levels in MFS patients compared with non-MFS patients. For the detection of MFS, 3D volumetry achieved higher AUCs at L3-S1 levels (0.743, 0.752, 0.808, and 0.824) compared with dural sac diameter ratios (0.673, 0.707, 0.791, and 0.848); a significant difference was observed only for L3 (*p* < 0.001). Conclusion: MRI-derived 3D volumetry of the lumbosacral dural sac and vertebral bodies is a feasible method for quantifying dural ectasia using shape-based machine learning. Non-inferior diagnostic accuracy was observed compared with dural sac diameter ratio (the current clinical standard for MFS detection).

## 1. Introduction

Marfan syndrome (MFS) is a hereditary connective tissue disorder that is transmitted in an autosomal dominant pattern and affects approximately 1 in 5000 individuals worldwide [1,2]. MFS can also occur sporadically in about 25% of cases as a new mutation in patients with no family history of the disease [3,4,5]. MFS is caused by mutations in the FBN 1 gene on chromosome 15 [6,7]. This leads to defective encoding of the glycoprotein fibrillin 1, a major component of connective tissue microfibrils [8], resulting in reduced stability and elasticity of connective tissue throughout the organism. The mutations on this single gene have a pleiotropic effect on different organ systems and lead to a high variability of clinical symptoms [9]. The cardiovascular system, skeleton, eyes, meninges, and lungs are particularly affected. The majority of symptoms develop later in life [10]. If left untreated, patients with MFS have a significantly reduced life expectancy, mainly due to cardiovascular complications such as aortic dissection [11]. Early diagnosis is essential to allow close monitoring and early prophylactic treatment, resulting in a life expectancy close to that of the general population [12]. The management of MFS today is similar to that of three decades ago [13]. On the one hand, early cardiosurgical implantation of an aortic valve and/or aortic prostheses is performed in cases of dilatation of the aortic root and aortic segments [14,15]. On the other hand, prophylactic treatment includes medical therapy with sartans and beta-blockers to prevent progressive aortic dilatation and aortic dissection [16,17]. A recent area of research is gene therapy, which may be able to alter the genotype of Marfan syndrome and thus prophylactically prevent the typical phenotype and associated complications [13]. Several therapeutic targets are currently in preclinical studies [13].

The diagnosis of MFS can be difficult due to variable phenotypic expression and is based on the 2010 Revised Ghent Nosology for MFS, which combines the assessment of two cardinal manifestations, family history, pathogenic mutations in the fibrillin-1 gene, and systemic manifestations [18]. The two cardinal manifestations of MFS are a dilated aortic root or aortic dissection [19,20] and ectopia lentis [21]. The Z-score is calculated for the presence of aortic root dilatation, which can be considered as the standard deviation of a normal value of the aortic root diameter at the sinus valsalva corrected for age, sex, height, and weight [22]. Dilatation of the aortic root can potentially lead to dissection, a serious and often life-threatening condition [19,20]. Ectopia lentis is a displacement or malposition of the crystalline lens of the eye, which can lead to visual impairment [21].

Imaging plays a crucial role in this diagnostic process, as it is used to assess the aortic root for dilatation and the lumbosacral spine for dural ectasia, one of the systemic manifestations of MFS that counts towards the systemic score of the 2010 Revised Ghent Nosology for MFS. Dural ectasia is described as expansion of the dural sac together with bone erosion of the vertebral bodies [23] or with expansion of the nerve root sheaths [24]. The dural sac and vertebral changes usually occur in the lumbosacral region of the spine, where the hydrostatic pressure of the cerebrospinal fluid is greatest in the upright patient [25]. They can present with genital, rectal and lower back pain, headache, proximal leg pain, and decreased sensation and strength above and below the knee [26,27], or even cause obstruction of other organs due to large meningoceles [28]. Dural ectasia occurs in a significant proportion of MFS patients, ranging from 63% to 92% [25,29]. Detection of dural ectasia is a critical component in the diagnosis of MFS and is necessary for making the diagnosis in up to 20% of MFS patients [30].

Magnetic resonance imaging (MRI) can provide a qualitative and quantitative morphological assessment of dural ectasia. Various MRI-based imaging criteria and cut-off values have been proposed to define dural ectasia, including the more commonly used criteria by Habermann et al. for children, adolescents, and young adults, and by Oosterhof et al. [29,31,32,33,34,35,36]. These criteria primarily include two-dimensional (2D) morphological parameters, in particular the dural sac diameter ratios of the lumbosacral spine. Other parameters include lumbar vertebral scalloping, herniation of nerve root sheaths, and anterior and lateral meningoceles. However, to date, no common standard has been established, and the use of different criteria and cut-off values depends largely on institutional preference.

The potential of three-dimensional (3D) parameters is being increasingly recognized. Three-dimensional, i.e., volumetric, parameters of dural ectasia may be more reliable than two-dimensional parameters, which are the current clinical standard. Furthermore, 3D information could be explored beyond volume to extract novel shape-based and radiomic features, which have been investigated, for example, in prostate cancer [37] or monoclonal plasma cell disorders [38]. However, as a first step, an MRI-derived 3D segmentation of the lumbosacral spine is required.

Automated segmentation using 3D statistical shape models has been shown to be robust and accurate [39]. The process involves placing a surface model in the image, followed by an iterative search for landmarks using local image information [40]. Despite the development of various automated segmentation methods, including heuristically customized appearance models and automatic selection of optimal local features [41], these methods are limited by their reliance on local image information and the use of heuristic or weak learning methods [40,42]. However, models utilizing randomized regression forests have emerged, offering robust learning that consider non-local image information and omni-directional landmark detection, thereby enhancing segmentation accuracy and robustness across various organs without the need for prior model initialization [40,43,44,45].

The rapid development of artificial intelligence has led to deep learning-based segmentation methods [46]. Deep learning, through deep neural networks, autonomously extracts data features, simulating the human brain’s learning process [46,47]. Convolutional neural networks, fully convolutional networks [46,48], U-Net networks [46,49], and further developments have been successfully applied to semantic image segmentation [50]. These methodologies represent a paradigm shift from traditional machine learning computer vision approaches with hand-crafted features and heuristic rules to deep learning-based techniques with automatic feature extraction and learning of hierarchical representations of data [51]. Among these, the nnU-Net network is of particular note for medical image segmentation [50,52], having the capability to automatically adapt to new datasets [53]. Recent years have seen various MRI-based segmentation studies for the spine covering different clinical questions. For example, Hohenhaus et al. developed an automated method for quantifying cervical spinal canal compromise using a deep convolutional neural network with U-Net architecture [54]. Van der Graaf et al. introduced a dataset for segmenting vertebrae, intervertebral discs, and spinal canal from MRI series, employing both an iterative data annotation approach and the nnU-Net algorithm, with both methods yielding nearly identical results [53]. Kim et al. demonstrated the use of 2D and 3D U-Nets for detecting bone metastasis [55], and Zhu et al. evaluated the 3D U-Net model for automatic segmentation and measurement of cervical spine structures, suggesting its potential for quantitative diagnosis models for spinal cord diseases [56]. Further studies have explored MRI-based deep learning segmentation of the spine, contributing to the field’s advancement [57,58,59,60,61,62,63]. However, to the best of our knowledge, application of 3D segmentation to the dural sac for the detection of MFS has not yet been published.

The aim of this study was to develop and assess the feasibility of MRI-derived 3D volumetry of the lower lumbar vertebrae and dural sac segments using shape-based machine learning. A further objective was to evaluate the diagnostic accuracy of 3D volumetry for the detection of MFS compared with dural sac diameter ratios, the current clinical standard.

## 2. Materials and Methods

### 2.1. Patients

This retrospective single-center study was approved by the institutional review board, and patient informed consent was waived. Records of all patients evaluated for MFS in the institutional center for MFS from January 2012 to December 2016 were reviewed. All patients undergoing MRI of the lumbosacral spine as part of the evaluation for MFS were included. Primary exclusion criteria were as follows: corrupt image data, motion artifacts, status post spondylodesis, and MRI showing undefined vertebral body contour (e.g., due to extensive degenerative changes). Secondary exclusion criteria were final diagnosis of a hereditary connective tissue disorder other than MFS, final diagnosis of MFS dependent on the lumbosacral MRI, and failure of automated 3D volumetry. Age, gender, height, weight, aortic root diameter, aortic disease, ectopia lentis, family history of MFS, fibrillin-1 gene mutations, and systemic manifestations of MFS were extracted from the records. The Z-score was calculated according to Devereux et al. [22], and the body surface area (BSA) according to the formula by Du Bois and Du Bois [64]. Diagnosis of MFS was made according to the 2010 Revised Ghent Nosology for Marfan syndrome independent of the lumbosacral MRI [18], resulting in two study groups: the MFS group and the non-MFS group.

### 2.2. Image Acquisition

MRI of the lumbosacral spine was performed using a 1.5 T MRI scanner (Aera, Siemens Healthineers, Erlangen, Germany) and a single slab 3D T2-weighted turbo spin echo sequence in coronal orientation without application of any contrast medium. Sequence parameters were as follows: spatial resolution 1 × 1 × 1 mm^3^, repetition time 1600 ms, and echo time 202 ms. Acquisition time was 5:06 min.

### 2.3. Image Analysis

Automated segmentation and volumetry of vertebral bodies L3-L5 and dural sac segments L3-S1 was performed applying a previously published shape-based machine learning algorithm [40,65]. This shape-based machine learning algorithm represents an extension of 3D statistical shape models. Whereas traditional 3D statistical shape models often rely on unidirectional search strategies that only consider local image information to find landmark positions, the extended algorithm also used in the present study incorporates landmark-wise random regression forests to perform an omni-directional search for landmark positions. This method takes advantage of rich non-local image information, which significantly enhances segmentation accuracy and precision. Furthermore, the algorithm applies a long-distance model fitting strategy based on a multi-scale approach, allowing for accurate and reproducible segmentation from distant image positions without the need for model initialization. An iterative model fitting is conducted to generate 3D shape models. The accuracy of the proposed omni-directional search and long-distance model fitting is demonstrated through segmentation experiments and competitive multi-organ labeling challenges, including liver, spleen, and kidney segmentation, with excellent results, e.g., for liver segmentation a volumetric overlap error of 5.90% and a volumetric difference of 1.17% [65].

In a first step, the algorithm automatically computed 3D segmentations of the vertebral bodies L3-L5 and the dural sac from above L3 to below S1. In a second step, the algorithm generated so-called clipping planes intersecting the intervertebral discs and thus defining the boundaries of the dural sac segments L3-S1.

The contour of the segmentation for the vertebral bodies generally followed the cortical substance. The segmentation excluded the vertebral arches by following the shortest distance between the posterior and the lateral surfaces of the vertebral bodies. Thus, the segmentation resulted in a characteristic cylindrical shape with concave lateral surfaces and oval- to bean-shaped transverse cross-sections. The segmentation of the dural sac followed the borders between the dural sac and bones or between the dural sac and the epidural space, respectively. Segmentation of a normal dural sac resulted in the shape of a tube with a ventrally convex curvature in the area of the lumbar lordosis and a dorsally convex curvature in the area of the sacral kyphosis, tapering conically towards the sacrum. The transverse cross-section of the dural sac showed primarily circular to oval shapes. 

**Figure 1:**
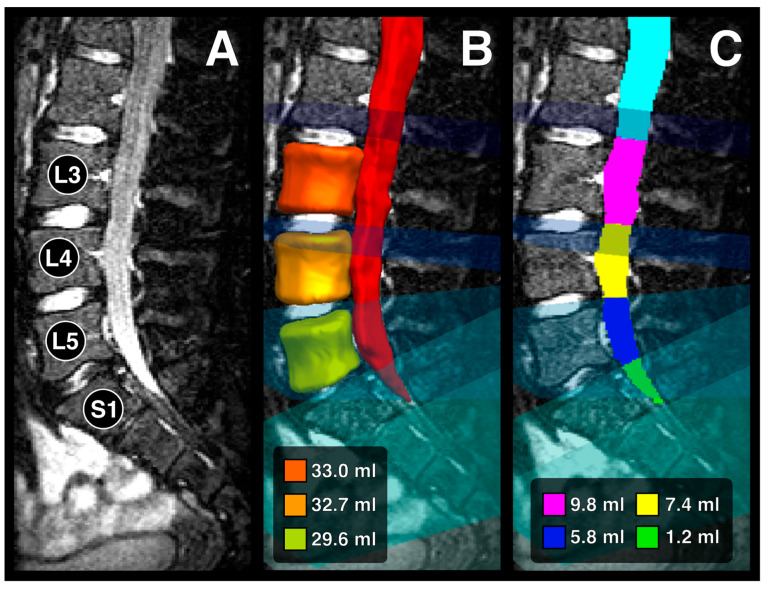
Representative example of a 3D segmentation of the lower lumbar vertebrae and dural sac segments in a non-MFS patient. (**A**) Sagittal view of the lumbosacral MRI. (**B**) Visualization of the 3D segmentation and volumes of the vertebrae. (**C**) Visualization of the intervertebral clipping planes and the resulting dural sac segments, including their volumes.

Segmentation results were visualized in the free open-source software The Medical Imaging Interaction Toolkit (MITK) Workbench (version 2016.11.0) (Figure 1). A radiology resident and a board-certified radiology consultant with 6 years of experience reviewed segmentations in consensus, and manually corrected visible deviations from the vertebral body and dural sac contours using the tools provided by the same software. The dural sac volume ratio (DSVR) for each level was calculated as the ratio between the dural sac volume and the vertebral body volume at the respective level. For Level S1, the volume ratio was computed as the ratio between the dural sac volume at level S1 and the vertebral body volume at level L5.

For comparison with the current clinical standard, evaluation of all lumbosacral MRI data according to clinical practice was conducted by one board-certified radiologist with dedicated experience in neuroradiology, blinded to any other measurements and clinical data. The following parameters were evaluated, as described in the literature [23]: vertebral body diameters for levels L3 to S1, dural sac diameters for levels L3 to S1, nerve root sleeve diameters at level L5, diameters of the upper and lower endplate levels of the vertebral body S1, and anterior sacral and lateral lumbosacral meningoceles. The dural sac diameter ratio (DSDR) for each level was calculated as the ratio between the dural sac diameter and the vertebral body diameter at the respective level as described in the literature [23]. Scalloping of S1 vertebral body was defined as the difference between vertebral body diameter S1 and the mean of its diameters of the upper and lower endplate levels as described in the literature [31].

### 2.4. Statistical Analysis

Continuous variables are described by mean ± standard deviation. Categorical variables are given as numbers and percentages. Receiver operating characteristic (ROC) analyses were performed to assess the ability to differentiate the MFS group from the non-MFS group patients, and the area under the curve (AUC) was calculated. Sensitivities, specificities, and positive and negative predictive values were calculated for the threshold values, yielding the maximum Youden index for each measurement. The McNemar test was applied to test for a statistical difference of classifications. A *p*-value of <0.05 was considered statistically significant. Statistical analyses were performed using SPSS version 27.0 (SPSS Inc., Chicago, IL, USA).

## 3. Results

### 3.1. Study Sample

**Figure 2:**
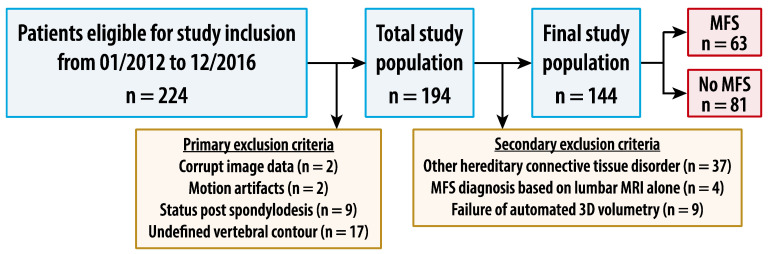
Flowchart of the study. MFS—Marfan syndrome.

A total of 224 patients fulfilled the inclusion criterion of undergoing MRI of the lumbosacral spine as part of the evaluation for MFS. A total of 30 patients were primarily excluded for the following reasons: corrupt image data (*n* = 2, 0.9%), motion artifacts (*n* = 2, 0.9%), status post spondylodesis (*n* = 9, 4.0%), and MRI showing undefined vertebral body contour (*n* = 17, 7.6%). A total of 50 patients were secondarily excluded for the following reasons: final diagnosis of a hereditary connective tissue disorder other than MFS (*n* = 37, 16.5%), final diagnosis of MFS dependent on the lumbosacral MRI (*n* = 4, 1.8%), and failure of automated 3D volumetry (*n* = 9, 4.0%). These exclusions resulted in a final study sample of 144 patients (Table 1). Figure 2 shows the flow chart of patient selection.

In the MFS group, 51 patients agreed to genetic testing, and 48 patients had a fibrillin-1 gene mutation pathogenic for Marfan syndrome. In the non-MFS group, 31 patients agreed to genetic testing, and 1 patient had a fibrillin-1 gene mutation of unclear significance. 

### 3.2. Volume Measurements

**Figure 3:**
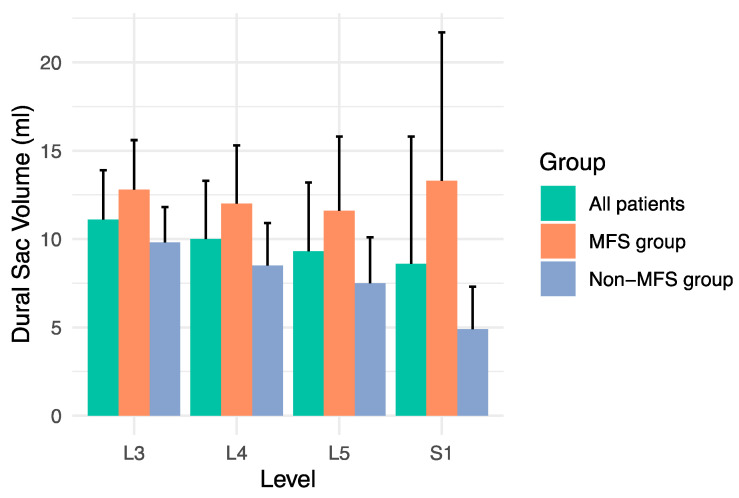
Dural sac volumes at measurement levels L3 to S1 for all patients, for patients with Marfan syndrome (MFS group), and for patients without Marfan syndrome (non-MFS group).

Dural sac volumes were significantly larger at all measurement levels in MFS patients compared with non-MFS patients (Figure 3). Means ± standard deviations in MFS vs. non-MFS patients were for level L3 12.8 ± 2.8 mL vs. 9.8 ± 2.0 mL (*p* < 0.001), for level L4 12.0 ± 3.3 mL vs. 8.5 ± 2.4 mL (*p* < 0.001), for level L5 11.6 ± 4.2 mL vs. 7.5 ± 2.6 mL (*p* < 0.001), and for level S1 13.3 ± 8.4 mL vs. 4.9 ± 2.4 mL (*p* < 0.001).

Dural sac volume ratios also were significantly higher at all measurement levels in MFS patients compared with non-MFS patients. Means ± standard deviations in MFS vs. non-MFS patients were for level L3 0.36 ± 0.10 vs. 0.28 ± 0.07 (*p* < 0.001), for level L4 0.33 ± 0.11 vs. 0.24 ± 0.07 (*p* < 0.001), for level L5 0.36 ± 0.14 vs. 0.22 ± 0.07 (*p* < 0.001), and for level S1 0.41 ± 0.28 vs. 0.14 ± 0.06 (*p* < 0.001).

Vertebral body volumes were not significantly different between MFS patients and non-MFS patients. Means ± standard deviations in MFS vs. non-MFS patients were for level L3 36.7 ± 7.4 mL vs. 36.6 ± 9.6 mL (*p* = 0.96), for level L4 37.4 ± 7.6 mL vs. 36.8 ± 8.9 mL (*p* = 0.71), and for level L5 33.2 ± 7.8 mL vs. 34.9 ± 7.8 mL (*p* = 0.21).

Corrected for body mass index (BMI), means ± standard deviations of dural sac volumes [in ml per kg/m^2^ BMI] in MFS vs. non-MFS patients were for level L3 0.58 ± 0.17 vs. 0.47 ± 0.14 (*p* < 0.001), for level L4 0.55 ± 0.19 vs. 0.42 ± 0.17 (*p* < 0.001), for level L5 0.53 ± 0.21 vs. 0.37 ± 0.17 (*p* < 0.001), and for level S1 0.59 ± 0.37 vs. 0.24 ± 0.14 (*p* < 0.001). 

Corrected for body mass index (BMI), means ± standard deviations of vertebral body volumes [in ml per kg/m^2^ BMI] in MFS vs. non-MFS patients were for level L3 1.64 ± 0.42 vs. 1.75 ± 0.46 (*p* = 0.15), for level L4 1.68 ± 0.45 vs. 1.78 ± 0.46 (*p* = 0.25), and for level L5 1.51 ± 0.44 vs. 1.69 ± 0.42 (*p* = 0.02).

Manual segmentation following automatic segmentation was necessary in six patients for the dural sac, of whom four were non-MFS patients and two were MFS patients, and in six patients for lumbar vertebrae, of whom five were non-MFS patients and one was an MFS patient. The average time needed for manual segmentation of the dural sac was 7.6 min per dural sac segment, and for manual segmentation of vertebrae was 16.4 min per vertebra. In this very small subset of patients with manual segmentations, the dural sac volumes, dural sac volume ratios, and vertebral body volumes were in the range of the values for the other patients, and the same tendencies between MFS vs. non-MFS patients were observed. In the cases regarding the dural sac, we observed very low image signal intensities of the dural sac, which likely affected the automatic segmentations. In the cases regarding the vertebrae, we observed anatomical abnormalities such as severe osteophytes, which likely affected the automatic segmentations. In these instances, full manual segmentations appeared to make more sense.

Detailed results of the volume measurements are shown in Supplementary Table S1.

### 3.3. Diameter Measurements

Dural sac diameters were significantly larger at all measurement levels in MFS patients compared with non-MFS patients. Means ± standard deviations in MFS vs. non-MFS patients were for level L1 18.6 ± 3.3 mm vs. 16.7 ± 2.3 mm (*p* < 0.001), for level L2 17.6 ± 2.1 mm vs. 15.7 ± 2.0 mm (*p* < 0.001), for level L3 17.0 ± 2.5 mm vs. 15.2 ± 1.9 mm (*p* < 0.001), for level L4 17.6 ± 3.0 mm vs. 14.5 ± 2.7 mm (*p* < 0.001), for level L5 19.3 ± 4.1 mm vs. 14.9 ± 3.0 mm (*p* < 0.001), and for level S1 19.1 ± 6.9 mm vs. 11.9 ± 3.3 mm (*p* < 0.001).

Dural sac diameter ratios also were significantly higher at all measurement levels in MFS patients compared with non-MFS patients. Means ± standard deviations in MFS vs. non-MFS patients were for level L1 0.67 ± 0.12 vs. 0.60 ± 0.11 (*p* = 0.003), for level L2 0.60 ± 0.11 vs. 0.54 ± 0.09 (*p* < 0.001), for level L3 0.55 ± 0.10 vs. 0.49 ± 0.09 (*p* = 0.002), for level L4 0.56 ± 0.11 vs. 0.48 ± 0.10 (*p* < 0.001), for level L5 0.66 ± 0.16 vs. 0.50 ± 0.12 (*p* < 0.001), and for level S1 1.11 ± 0.68 vs. 0.50 ± 0.14 (*p* < 0.001).

L5 nerve root sleeve diameter was not significantly different between MFS patients and non-MFS patients. Mean ± standard deviation in MFS vs. non-MFS patients was 6.1 ± 1.2 mm vs. 6.0 ± 1.0 mm (*p* = 0.64).

S1 scalloping was significantly increased in MFS patients compared with non-MFS patients. Mean ± standard deviation in MFS vs. non-MFS patients was 5.0 ± 2.5 mm vs. 3.1 ± 1.3 mm (*p* < 0.001).

Vertebral body diameters were mostly not significantly different between MFS patients and non-MFS patients. Means ± standard deviations in MFS vs. non-MFS patients were for level L1 28.6 ± 5.8 mm vs. 28.0 ± 3.8 mm (*p* = 0.49), for level L2 30.1 ± 4.3 mm vs. 29.6 ± 3.6 mm (*p* = 0.52), for level L3 31.5 ± 3.7 mm vs. 31.1 ± 3.7 mm (*p* = 0.48), for level L4 31.7 ± 3.3 mm vs. 31.2 ± 3.6 mm (*p* = 0.40), for level L5 29.5 ± 3.4 mm vs. 30.2 ± 3.1 mm (*p* = 0.18), and for level S1 19.3 ± 4.4 mm vs. 23.8 ± 3.2 mm (*p* < 0.001).

Corrected for body mass index (BMI), means ± standard deviations of dural sac diameters [in mm per kg/m^2^ BMI] in MFS vs. non-MFS patients were for level L1 0.86 ± 0.23 vs. 0.79 ± 0.19 (*p* = 0.14), for level L2 0.81 ± 0.16 vs. 0.75 ± 0.17 (*p* = 0.08), for level L3 0.78 ± 0.18 vs. 0.74 ± 0.19 (*p* = 0.30), for level L4 0.80 ± 0.20 vs. 0.71 ± 0.22 (*p* = 0.016), for level L5 0.88 ± 0.24 vs. 0.73 ± 0.23 (*p* = 0.001), and for level S1 0.85 ± 0.33 vs. 0.58 ± 0.21 (*p* < 0.001).

Corrected for body mass index (BMI), means ± standard deviations of L5 nerve root sleeve diameter [in mm per kg/m^2^ BMI] in MFS vs. non-MFS patients was 0.28 ± 0.07 vs. 0.29 ± 0.06 (*p* = 0.25), and means ± standard deviations of S1 scalloping [in mm per kg/m^2^ BMI] in MFS vs. non-MFS patients was 0.22 ± 0.12 vs. 0.15 ± 0.07 (*p* < 0.001).

Corrected for body mass index (BMI), means ± standard deviations of vertebral body diameters [in mm per kg/m^2^ BMI] in MFS vs. non-MFS patients were for level L1 1.31 ± 0.37 vs. 1.32 ± 0.28 (*p* = 0.84), for level L2 1.37 ± 0.25 vs. 1.43 ± 0.28 (*p* = 0.21), for level L3 1.43 ± 0.27 vs. 1.50 ± 0.30 (*p* = 0.30), for level L4 1.44 ± 0.27 vs. 1.52 ± 0.32 (*p* = 0.14), for level L5 1.35 ± 0.27 vs. 1.46 ± 0.26 (*p* = 0.016), and for level S1 0.90 ± 0.25 vs. 1.16 ± 0.25 (*p* < 0.001).

Detailed results of the diameter measurements are shown in Supplementary Table S2. 

### 3.4. Identification of Marfan Syndrome Patients

**Figure 4:**
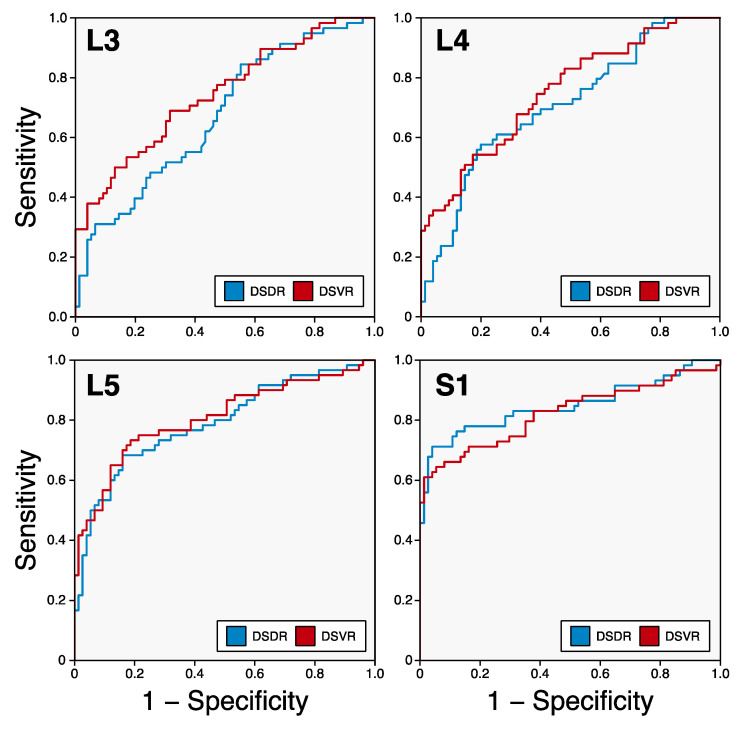
ROC curves for the detection of Marfan syndrome. DSDR—dural sac diameter ratio, DSVR—dural sac volume ratio.

In ROC analysis, the parameters with the highest AUC for prediction of MFS were S1 volume ratio (0.824; 95% confidence interval 0.746, 0.901) and S1 diameter ratio (0.848; 95% confidence interval 0.775, 0.922) with no significant difference (*p* = 0.18, Table 2). For S1 volume ratio/S1 diameter ratio, diagnostic performance using a cut-off value of ≥0.25/≥0.75, respectively, was as follows: sensitivity 62%/71%, specificity 97%/96%, positive predictive value 97%/94% and negative predictive value 57%/81%, and false positive rate 3%/4%. For the measurement levels L3-L5, AUCs for volume ratios were higher compared with those for diameter ratios (Figure 4, Table 2).

## 4. Discussion

MRI-derived 3D segmentation of the lower lumbar vertebrae and dural sac segments using shape-based machine learning is feasible and demonstrates significantly increased dural sac volumes and dural sac volume ratios for levels L3 to S1 in patients with MFS compared with non-MFS controls. Furthermore, dural sac volume ratios tend to have higher diagnostic accuracy for detecting MFS compared with manually measured dural sac diameter ratios, although the difference is small and not clinically relevant.

These findings are well in accordance with numerous previous studies investigating lumbosacral dural sac and vertebral body diameters. The relationship between MFS and dural ectasia has been well documented, and patients with MFS have been shown to exhibit significantly enlarged lumbosacral dural sac diameters and ratios [23,30,31,32,33,34,36,66,67,68,69]. Furthermore, the sensitivity, specificity, and positive and negative predictive values found in our study were in the range established by previous studies [31,32,33,34,66,68]. 

Of note, mean dural sac diameter ratios in the current study were higher compared with values reported in several previous studies with comparable patient populations [32,34,66,69]. Consecutively, the optimal cut-off value to differentiate MFS from non-MFS patients was also higher in the present study [33,34]. This may be explained by the use of different higher resolution MRI sequences in the present study compared with previous studies and differences between the study populations, e.g., regarding patient age and the nature of the control group. For example, children and adolescents have a lower prevalence of dural ectasia than adults [30,32,70]. Moreover, previous studies mainly were case-control studies with controls referred to MRI for entirely different indications, e.g., back pain [32,34]. However, this observation underlines the importance of institution-specific reference and cut-off values to avoid any systematic bias introduced by local peculiarities of the MRI protocol and the patient population and to ensure consistent diagnostic accuracy.

Our findings corroborate these previous studies, adding a novel dimension by quantifying dural sac volumes and examining their diagnostic utility. A key aspect of our study is the application of 3D volumetry, which could potentially address the limitations of 2D measurements that may not fully capture the spatial complexities of dural ectasia. In the present study, we demonstrate feasibility of MRI-derived 3D segmentation of the lower lumbar vertebrae and dural sac segments. The potential of 3D segmentation goes beyond the calculation of volumes. First, radiomic features can be computed based on the 3D segmentations, such as curvature and surface properties of the shape models. Radiomic features, which are quantitative descriptors extracted from medical images, could provide insights into disease characteristics that are not appreciable through visual inspection alone. Machine learning algorithms can process these features to identify patterns associated with specific conditions, potentially leading to improved diagnostic and prognostic models. Second, the information from the patient-specific fitting of the statistical shape model can be quantitatively evaluated and in turn yields its own quantitative parameters that can be used to differentiate between MFS and non-MFS patients. The use of extracted radiomic features from 3D segmentations of imaging data is already being explored, for example, in lung carcinoma [71,72], prostate carcinoma [37], skin lesion detection and classification [73], monoclonal plasma cell disorders [38], and anatomic studies [74]. However, this goes beyond this project and is the subject of further work. In principle, the 3D segmentation method is independent of the studied patient population, i.e., the method could also be applied to other study populations and questions.

Future research should focus on the validation of the segmentation algorithm in larger multicentric cohorts to establish generalizability. Furthermore, the exploration of additional radiomic features and the use of machine learning for pattern recognition could enhance our understanding of MFS and improve diagnostic workflows. The application of 3D volumetry and machine learning to other hereditary connective tissue disorders could also be beneficial. By comparing the morphological features across different conditions, we may be able to better differentiate between phenotypically similar syndromes and tailor management strategies accordingly.

While our study provides promising results, it is not without limitations. First, it has to be mentioned that there is no single test that is the gold standard for the diagnosis of MFS; rather, the true diagnosis relies on the comprehensive assessment of the diagnostic criteria according to the 2010 Ghent Nosology, which itself is a composite of clinical findings and genetic testing. This composite nature of the diagnostic criteria may introduce a degree of subjectivity and variability in the diagnosis. 

Additionally, the accuracy of the segmentation algorithm is not independently validated within this study. While the consensus review by experts adds a layer of validation, a formal assessment against a gold standard or through phantom studies would provide a more robust evaluation of the algorithm’s performance. The present study focuses on establishing the feasibility and evaluation of whether 3D quantification yields meaningful results. Furthermore, validation in an in vivo setting is always subject to limitations due to the lack of a gold standard. Nonetheless, validation regarding other related MRI datasets is desirable.

Finally, in recent years, there have been notable developments in artificial intelligence, particularly in the areas of deep learning and computational capabilities [51]. These advancements have led to improvements in image segmentation methods, which now demonstrate enhanced performance over traditional machine learning and computer vision techniques in aspects of segmentation quality and efficiency [51]. Consequently, it would be worthwhile to explore the application of contemporary deep learning segmentation methods to our datasets. This could also potentially reduce the need for manual correction.

In conclusion, MRI-derived 3D volumetry of lumbosacral dural sac and vertebral bodies for quantifying dural ectasia using shape-based machine learning is feasible and shows non-inferior diagnostic accuracy for detecting MFS compared with 2D dural sac diameter ratios as the current clinical standard. The potential for further refinement of this technique through radiomics and utilization of the information from the patient-specific fitting may lead to even greater diagnostic precision, ultimately improving patient outcomes in MFS and potentially other connective tissue disorders. Further research is warranted to validate these findings and explore the full clinical utility of this approach.

## Figures and Tables

**Table 1:** Clinical characteristics of the study sample.

	Non-MFS Group (*n* = 81)	MFS Group (*n* = 63)	*p*-Value
Sex			0.97
Female	46 (57%)	36 (57%)	
Male	35 (43%)	27 (43%)	
Age (years)	36 ± 16	35 ± 11	0.81
Height (cm)	182.0 ± 9.8	187.8 ± 10.6	0.002
Weight (kg)	70.8 ± 15.5	79.4 ± 13.7	0.002
BSA (m^2^)	1.90 ± 0.22	2.05 ± 0.21	<0.001
BMI (kg/m^2^)	21.3 ± 4.1	22.5 ± 3.2	0.07
Aortic root diameter (cm)	3.3 ± 0.6	4.2 ± 0.7	<0.001
Z score	1.6 ± 1.7	3.7 ± 2.5	<0.001

Values are presented as mean ± SD, number of patients with percentage in parentheses. BSA—body surface area. BMI—body mass index.

**Table 2:** ROC analysis of volume ratios and diameter ratios to differentiate Marfan syndrome patients from non-Marfan syndrome patients.

	Volume Ratio	Diameter Ratio	*p*-Value
L1	-	0.603 (0.506, 0.700)	-
L2	-	0.636 (0.545, 0.728)	-
L3	0.743 (0.659, 0.828)	0.673 (0.582, 0.764)	<0.001
L4	0.752 (0.670, 0.834)	0.707 (0.619, 0.795)	0.12
L5	0.808 (0.730, 0.885)	0.791 (0.713, 0.870)	0.30
S1	0.824 (0.746, 0.901)	0.848 (0.775, 0.922)	0.18

Values are given as area under the curve (95% confidence interval).

## Data Availability

All relevant data are within the paper. Please contact the corresponding author for further requests.

## Supplementary Materials

The following supporting information can be downloaded at: https://www.mdpi.com/article/10.3390/diagnostics14121301/s1, Table S1: Detailed results of volume measurements; Table S2: Detailed results of diameter measurements.

## References

[1] McKusick V.A. (1955). The cardiovascular aspects of Marfan’s syndrome: A heritable disorder of connective tissue. Circulation.

[2] Weve H. (1931). Über Arachnodaktylie (Dystrophia mesodermalis congenita, Typus Marfan). Arch. Augenheilk..

[3] Gray J.R., Bridges A.B., Faed M.J., Pringle T., Baines P., Dean J., Boxer M. (1994). Ascertainment and severity of Marfan syndrome in a Scottish population. J. Med. Genet..

[4] Pyeritz R.E. (1986). The Marfan syndrome. Am. Fam. Physician.

[5] Marfan A.B.J. (1896). Un Cas de Déformation Congénitale des Quatre Membres, Plus Prononcée aux Extrémités, Caractérisée par L’allongement des Os avec un Certain Degré D’amincissement.

[6] Dietz H.C., Cutting G.R., Pyeritz R.E., Maslen C.L., Sakai L.Y., Corson G.M., Puffenberger E.G., Hamosh A., Nanthakumar E.J., Curristin S.M. (1991). Marfan syndrome caused by a recurrent de novo missense mutation in the fibrillin gene. Nature.

[7] Dietz H.C., Pyeritz R.E., Hall B.D., Cadle R.G., Hamosh A., Schwartz J., Meyers D.A., Francomano C.A. (1991). The Marfan syndrome locus: Confirmation of assignment to chromosome 15 and identification of tightly linked markers at 15q15-q21.3. Genomics.

[8] Sakai L.Y., Keene D.R., Engvall E. (1986). Fibrillin, a new 350-kD glycoprotein, is a component of extracellular microfibrils. J. Cell Biol..

[9] Pyeritz R.E. (1989). Pleiotropy revisited: Molecular explanations of a classic concept. Am. J. Med. Genet..

[10] Lipscomb K.J., Clayton-Smith J., Harris R. (1997). Evolving phenotype of Marfan’s syndrome. Arch. Dis. Child..

[11] Murdoch J.L., Walker B.A., Halpern B.L., Kuzma J.W., McKusick V.A. (1972). Life expectancy and causes of death in the Marfan syndrome. N. Engl. J. Med..

[12] Pyeritz R.E. (2017). Etiology and pathogenesis of the Marfan syndrome: Current understanding. Ann. Cardiothorac. Surg..

[13] Kallenbach K., Remes A., Müller O.J., Arif R., Zaradzki M., Wagner A.H. (2022). Translational Medicine: Towards Gene Therapy of Marfan Syndrome. J. Clin. Med..

[14] Gott V.L., Cameron D.E., Pyeritz R.E., Gillinov A.M., Greene P.S., Stone C.D., Alejo D.E., McKusick V.A. (1994). Composite graft repair of Marfan aneurysm of the ascending aorta: Results in 150 patients. J. Card. Surg..

[15] Svensson L.G., Crawford E.S., Coselli J.S., Safi H.J., Hess K.R. (1989). Impact of cardiovascular operation on survival in the Marfan patient. Circulation.

[16] Child A., Stuart A.G., Aragon-Martin J.A., Yuan L., Hu J., Van Dyck L., Knight R., Clayton T., Flather M., Dean J. (2019). Irbesartan in Marfan syndrome (AIMS): A double-blind, placebo-controlled randomised trial. Lancet (Br. Ed.).

[17] Shores J., Berger K.R., Murphy E.A., Pyeritz R.E. (1994). Progression of aortic dilatation and the benefit of long-term beta-adrenergic blockade in Marfan’s syndrome. N. Engl. J. Med..

[18] Loeys B.L., Dietz H.C., Braverman A.C., Callewaert B.L., De Backer J., Devereux R.B., Hilhorst-Hofstee Y., Jondeau G., Faivre L., Milewicz D.M. (2010). The revised Ghent nosology for the Marfan syndrome. J. Med. Genet..

[19] Baer R.W., Taussig H.B., Oppenheimer E.H. (1943). Congenital aneurysmal dilatation of the aorta associated with arachnodactyly. Bull. Johns Hopkins Hosp..

[20] Etter L.E., Glover L.P. (1943). Arachnodactyly complicated by dislocated lens and death from rupture of dissecting aneurysm of aorta. JAMA.

[21] Borger F. (1914). Über zwei Fälle von Arachnodaktylie. Z. Kinderheilkd..

[22] Devereux R.B., de Simone G., Arnett D.K., Best L.G., Boerwinkle E., Howard B.V., Kitzman D., Lee E.T., Mosley T.H., Weder A. (2012). Normal limits in relation to age, body size and gender of two-dimensional echocardiographic aortic root dimensions in persons ≥15 years of age. Am. J. Cardiol..

[23] Sheikhzadeh S., Sondermann C., Rybczynski M., Habermann C.R., Brockstaedt L., Keyser B., Kaemmerer H., Mir T., Staebler A., Robinson P.N. (2014). Comprehensive analysis of dural ectasia in 150 patients with a causative FBN1 mutation. Clin. Genet..

[24] Mesfin A., Ahn N.U., Carrino J.A., Sponseller P.D. (2013). Ten-year clinical and imaging follow-up of dural ectasia in adults with Marfan syndrome. Spine J..

[25] Pyeritz R.E., Fishman E.K., Bernhardt B.A., Siegelman S.S. (1988). Dural ectasia is a common feature of the Marfan syndrome. Am. J. Hum. Genet..

[26] Ahn N.U., Sponseller P.D., Ahn U.M., Nallamshetty L., Kuszyk B.S., Zinreich S.J. (2000). Dural ectasia is associated with back pain in Marfan syndrome. Spine.

[27] Foran J.R., Pyeritz R.E., Dietz H.C., Sponseller P.D. (2005). Characterization of the symptoms associated with dural ectasia in the Marfan patient. Am. J. Med. Genet. A.

[28] Begley K., Sergides Y. (2022). Giant sacral dural ectasia causing ureteric obstruction in Marfan syndrome. ANZ J. Surg..

[29] Fattori R., Nienaber C.A., Descovich B., Ambrosetto P., Reggiani L.B., Pepe G., Kaufmann U., Negrini E., von Kodolitsch Y., Gensini G.F. (1999). Importance of dural ectasia in phenotypic assessment of Marfan’s syndrome. Lancet.

[30] Attanasio M., Pratelli E., Porciani M.C., Evangelisti L., Torricelli E., Pellicano G., Abbate R., Gensini G.F., Pepe G. (2013). Dural ectasia and FBN1 mutation screening of 40 patients with Marfan syndrome and related disorders: Role of dural ectasia for the diagnosis. Eur. J. Med. Genet..

[31] Ahn N.U., Sponseller P.D., Ahn U.M., Nallamshetty L., Rose P.S., Buchowski J.M., Garrett E.S., Kuszyk B.S., Fishman E.K., Zinreich S.J. (2000). Dural ectasia in the Marfan syndrome: MR and CT findings and criteria. Genet. Med..

[32] Habermann C.R., Weiss F., Schoder V., Cramer M.C., Kemper J., Wittkugel O., Adam G. (2005). MR evaluation of dural ectasia in Marfan syndrome: Reassessment of the established criteria in children, adolescents, and young adults. Radiology.

[33] Lundby R., Rand-Hendriksen S., Hald J.K., Lilleas F.G., Pripp A.H., Skaar S., Paus B., Geiran O., Smith H.J. (2009). Dural ectasia in Marfan syndrome: A case control study. Am. J. Neuroradiol..

[34] Oosterhof T., Groenink M., Hulsmans F.J., Mulder B.J., van der Wall E.E., Smit R., Hennekam R.C. (2001). Quantitative assessment of dural ectasia as a marker for Marfan syndrome. Radiology.

[35] Soylen B., Hinz K., Prokein J., Becker H., Schmidtke J., Arslan-Kirchner M. (2009). Performance of a new quantitative method for assessing dural ectasia in patients with FBN1 mutations and clinical features of Marfan syndrome. Neuroradiology.

[36] Villeirs G.M., Van Tongerloo A.J., Verstraete K.L., Kunnen M.F., De Paepe A.M. (1999). Widening of the spinal canal and dural ectasia in Marfan’s syndrome: Assessment by CT. Neuroradiology.

[37] Zhang K.S., Schelb P., Kohl S., Radtke J.P., Wiesenfarth M., Schimmoller L., Kuder T.A., Stenzinger A., Hohenfellner M., Schlemmer H.-P. (2021). Improvement of PI-RADS-dependent prostate cancer classification by quantitative image assessment using radiomics or mean ADC. Magn. Reson. Imaging.

[38] Wennmann M., Bauer F., Klein A., Chmelik J., Grözinger M., Rotkopf L.T., Neher P., Gnirs R., Kurz F.T., Nonnenmacher T. (2023). In vivo repeatability and multiscanner reproducibility of MRI radiomics features in patients with monoclonal plasma cell disorders: A prospective bi-institutional study. Investig. Radiol..

[39] Heimann T., Meinzer H.P. (2009). Statistical shape models for 3D medical image segmentation: A review. Med. Image Anal..

[40] Norajitra T., Engelhardt S., Held T., Al-Maisary S., de Simone R., Meinzer H.-P., Maier-Hein K. (2016). Statistische 3D-Formmodelle mit verteilter Erscheinungsmodellierung. Bildverarbeitung für die Medizin 2016.

[41] Kainmüller D., Lange T., Lamecker H. Shape constrained automatic segmentation of the liver based on a heuristic intensity model. Proceedings of the MICCAI Workshop 3D Segmentation in the Clinic.

[42] Ginneken B.V., Frangi A.F., Staal J.J. (2002). Active shape model segmentation with optimal features. IEEE Trans. Med. Imaging.

[43] Norajitra T., Meinzer H.P., Maier-Hein K.H. 3D statistical shape models incorporating random regression forest voting for multi-organ segmentation. Proceedings of the MICCAI 2015.

[44] Norajitra T., Meinzer H.-P., Maier-Hein K. 3D Regression Voting on CT-Volumes of the Human Liver for SSM Surface Appearance Modeling. Proceedings of the Shape 2014—Symposium on Statistical Shape Models and Applications.

[45] Norajitra T., Meinzer H.-P., Maier-Hein K. 3D statistical shape models incorporating 3D random forest regression voting for robust CT liver segmentation. Proceedings of the SPIE Medical Imaging.

[46] Liu X., Song L., Liu S., Zhang Y. (2021). A Review of Deep-Learning-Based Medical Image Segmentation Methods. Sustainability.

[47] Krizhevsky A., Sutskever I., Hinton G.E. (2012). ImageNet classification with deep convolutional neural networks. Commun. ACM.

[48] Long J., Shelhamer E., Darrell T. Fully convolutional networks for semantic segmentation. Proceedings of the IEEE Conference on Computer Vision and Pattern Recognition.

[49] Ronneberger O., Fischer P., Brox T. U-net: Convolutional networks for biomedical image segmentation. Proceedings of the Medical Image Computing and Computer-Assisted Intervention—MICCAI 2015: 18th International Conference.

[50] Isensee F., Jaeger P.F., Kohl S.A., Petersen J., Maier-Hein K.H. (2021). nnU-Net: A self-configuring method for deep learning-based biomedical image segmentation. Nature methods.

[51] Rayed M.E., Islam S.S., Niha S.I., Jim J.R., Kabir M.M., Mridha M. (2024). Deep learning for medical image segmentation: State-of-the-art advancements and challenges. Inform. Med. Unlocked.

[52] Isensee F., Petersen J., Klein A., Zimmerer D., Jaeger P.F., Kohl S., Wasserthal J., Koehler G., Norajitra T., Wirkert S. (2018). nnu-net: Self-adapting framework for u-net-based medical image segmentation. arXiv.

[53] van der Graaf J.W., van Hooff M.L., Buckens C.F., Rutten M., van Susante J.L., Kroeze R.J., de Kleuver M., van Ginneken B., Lessmann N. (2024). Lumbar spine segmentation in MR images: A dataset and a public benchmark. Sci. Data.

[54] Hohenhaus M., Klingler J.-H., Scholz C., Watzlawick R., Hubbe U., Beck J., Reisert M., Würtemberger U., Kremers N., Wolf K. (2024). Quantification of cervical spinal stenosis by automated 3D MRI segmentation of spinal cord and cerebrospinal fluid space. Spinal Cord.

[55] Kim D.H., Seo J., Lee J.H., Jeon E.-T., Jeong D., Chae H.D., Lee E., Kang J.H., Choi Y.-H., Kim H.J. (2024). Automated Detection and Segmentation of Bone Metastases on Spine MRI Using U-Net: A Multicenter Study. Korean J. Radiol..

[56] Zhu Y., Li Y., Wang K., Li J., Zhang X., Zhang Y., Li J., Wang X. (2024). A quantitative evaluation of the deep learning model of segmentation and measurement of cervical spine MRI in healthy adults. J. Appl. Clin. Med. Phys..

[57] Rak M., Steffen J., Meyer A., Hansen C., Tönnies K.D. (2019). Combining convolutional neural networks and star convex cuts for fast whole spine vertebra segmentation in MRI. Comput. Methods Programs Biomed..

[58] Kolarik M., Burget R., Riha K., Bartusek K. Suitability of CT and MRI Imaging for Automatic Spine Segmentation Using Deep Learning. Proceedings of the 2021 44th International Conference on Telecommunications and Signal Processing (TSP).

[59] Li H., Luo H., Huan W., Shi Z., Yan C., Wang L., Mu Y., Liu Y. (2021). Automatic lumbar spinal MRI image segmentation with a multi-scale attention network. Neural Comput. Appl..

[60] Wang S., Jiang Z., Yang H., Li X., Yang Z. (2022). Automatic segmentation of lumbar spine MRI images based on improved attention U-net. Comput. Intell. Neurosci..

[61] Cai B., Xu Q., Yang C., Lu Y., Ge C., Wang Z., Liu K., Qiu X., Chang S. (2023). Spine MRI image segmentation method based on ASPP and U-Net network. Math. Biosci. Eng..

[62] He S., Li Q., Li X., Zhang M. (2023). An optimized segmentation convolutional neural network with dynamic energy loss function for 3D reconstruction of lumbar spine MR images. Comput. Biol. Med..

[63] Li L., Qin J., Lv L., Cheng M., Wang B., Xia D., Wang S. (2023). ICUnet++: An Inception-CBAM network based on Unet++ for MR spine image segmentation. Int. J. Mach. Learn. Cybern..

[64] Du Bois D., Du Bois E.F. (1916). Clinical Calorimetry: Tenth Paper a Formula to Estimate the Approximate Surface Area If Height and Weight Be Known. Arch. Intern. Med..

[65] Norajitra T., Maier-Hein K.H. (2017). 3D Statistical Shape Models Incorporating Landmark-Wise Random Regression Forests for Omni-Directional Landmark Detection. IEEE Trans. Med. Imaging.

[66] Böker T., Vanem T.T., Pripp A.H., Rand-Hendriksen S., Paus B., Smith H.-J., Lundby R. (2019). Dural ectasia in Marfan syndrome and other hereditary connective tissue disorders: A 10-year follow-up study. Spine J..

[67] Rand-Hendriksen S., Lundby R., Tjeldhorn L., Andersen K., Offstad J., Semb S.O., Smith H.J., Paus B., Geiran O. (2009). Prevalence data on all Ghent features in a cross-sectional study of 87 adults with proven Marfan syndrome. Eur. J. Hum. Genet..

[68] Sznajder M., Krug P., Taylor M., Moura B., Leparc J.M., Boileau C., Jondeau G., Chevallier B., Pelage J.P., Stheneur C. (2010). Spinal imaging contributes to the diagnosis of Marfan syndrome. Jt. Bone Spine.

[69] Weigang E., Ghanem N., Chang X.C., Richter H., Frydrychowicz A., Szabo G., Dudeck O., Knirsch W., von Samson P., Langer M. (2006). Evaluation of three different measurement methods for dural ectasia in Marfan syndrome. Clin. Radiol..

[70] Knirsch W., Kurtz C., Haffner N., Binz G., Heim P., Winkler P., Baumgartner D., Freund-Unsinn K., Stern H., Kaemmerer H. (2006). Dural ectasia in children with Marfan syndrome: A prospective, multicenter, patient-control study. Am. J. Med. Genet. A.

[71] Liu Q., Huang Y., Chen H., Liu Y., Liang R., Zeng Q. (2020). Computed Tomography-Based Radiomic Features for Diagnosis of Indeterminate Small Pulmonary Nodules. J. Comput. Assist. Tomogr..

[72] Zhou H., Dong D., Chen B., Fang M., Cheng Y., Gan Y., Zhang R., Zhang L., Zang Y., Liu Z. (2018). Diagnosis of Distant Metastasis of Lung Cancer: Based on Clinical and Radiomic Features. Transl. Oncol..

[73] Debelee T.G. (2023). Skin Lesion Classification and Detection Using Machine Learning Techniques: A Systematic Review. Diagnostics.

[74] Stabile A.M., Pistilli A., Mariangela R., Rende M., Bartolini D., Di Sante G. (2023). New Challenges for Anatomists in the Era of Omics. Diagnostics.

