# Supplementary material for: MRI-Derived Dural Sac and Lumbar Vertebrae 3D Volumetry Has Potential for Detection of Marfan Syndrome
Source: Diagnostics (Basel). 2024 Jun 19;14(12):1301. doi: 10.3390/diagnostics14121301 (PMC11202825; doi:10.3390/diagnostics14121301)
Supplement: Supplementary file 1 [file diagnostics-14-01301-s001.zip › diagnostics-2879205-supplementary.pdf]

Table S1. Detailed results of volume measurements.

	All patients (n = 144)	Non-MFS group (n = 81)	MFS group (n = 63)	P-value
**Vertebral body volume (ml)**				
**L3**	36.6 ± 8.7 (23.8, 54.1)	36.6 ± 9.6 (23.9, 58.7)	36.7 ± 7.4 (24.1, 48.8)	0.96
**L4**	37.1 ± 8.3 (23.7, 52.9)	36.8 ± 8.9 (24.0, 55.7)	37.4 ± 7.6 (23.4, 49.2)	0.71
**L5**	34.2 ± 7.9 (21.6, 52.8)	34.9 ± 7.8 (23.3, 53.2)	33.2 ± 7.8 (19.8, 50.2)	0.21
**Dural sac volume (ml)**				
**L3**	11.1 ± 2.8 (7.0, 16.9)	9.8 ± 2.0 (6.7, 14.9)	12.8 ± 2.8 (8.6, 17.6)	<0.001
**L4**	10.0 ± 3.3 (5.5, 16.9)	8.5 ± 2.4 (5.4, 14.4)	12.0 ± 3.3 (6.4, 17.8)	<0.001
**L5**	9.3 ± 3.9 (3.6, 17.8)	7.5 ± 2.6 (3.6, 13.3)	11.6 ± 4.2 (4.1, 19.7)	<0.001
**S1**	8.6 ± 7.2 (1.1, 26.8)	4.9 ± 2.4 (1.2, 9.3)	13.3 ± 8.4 (1.4, 28.5)	<0.001
**Volume ratio**				
**L3**	0.31 ± 0.09 (0.18, 0.53)	0.28 ± 0.07 (0.16, 0.40)	0.36 ± 0.10 (0.21, 0.55)	<0.001
**L4**	0.28 ± 0.10 (0.14, 0.50)	0.24 ± 0.07 (0.13, 0.38)	0.33 ± 0.11 (0.18, 0.52)	<0.001
**L5**	0.28 ± 0.12 (0.12, 0.57)	0.22 ± 0.07 (0.11, 0.36)	0.36 ± 0.14 (0.13, 0.64)	<0.001
**S1***	0.26 ± 0.24 (0.03, 0.86)	0.14 ± 0.06 (0.03, 0.27)	0.41 ± 0.28 (0.04, 1.0)	<0.001

Data are mean ± standard deviation (95% confidence interval). MFS = Marfan syndrome. *Volume ratio for level S1 calculated as the ratio between the dural sac volume at level S1 and the vertebral body volume at level L5.

Table S2. Detailed results of diameter measurements.

	All patients (n=144)	Non-MFS group (n=81)	MFS group (n=63)	P-value
**Vertebral body diameter (mm)**				
**L1**	28.3 ± 4.8 (22.0, 37.4)	28.0 ± 3.8 (22.2, 35.7)	28.6 ± 5.8 (21.9, 37.9)	0.49
**L2**	29.8 ± 3.9 (24.2, 37.1)	29.6 ± 3.6 (24.0, 36.3)	30.1 ± 4.3 (24.6, 40.7)	0.52
**L3**	31.3 ± 3.7 (25.7, 40.2)	31.1 ± 3.7 (25.4, 40.0)	31.5 ± 3.7 (25.9, 40.2)	0.48
**L4**	31.4 ± 3.5 (25.9, 38.8)	31.2 ± 3.6 (25.6, 38.8)	31.7 ± 3.3 (26.5, 38.0)	0.40
**L5**	29.9 ± 3.3 (24.9, 37.2)	30.2 ± 3.1 (25.4, 36.6)	29.5 ± 3.4 (24.4, 37.1)	0.18
**S1**	21.8 ± 4.4 (11.8, 28.8)	23.8 ± 3.2 (19.0, 30.8)	19.3 ± 4.4 (10.0, 26.6)	<0.001
**Dural sac diameter (mm)**				
**L1**	17.6 ± 2.9 (12.6, 21.9)	16.7 ± 2.3 (11.3, 20.7)	18.6 ± 3.3 (13.9, 25.2)	<0.001
**L2**	16.6 ± 2.3 (12.8, 21.2)	15.7 ± 2.0 (12.5, 19.9)	17.6 ± 2.1 (14.3, 21.6)	<0.001
**L3**	16.0 ± 2.4 (12.0, 20.7)	15.2 ± 1.9 (11.6, 18.7)	17.0 ± 2.5 (12.7, 21.6)	<0.001
**L4**	15.9 ± 3.2 (10.7, 22.0)	14.5 ± 2.7 (9.5, 20.0)	17.6 ± 3.0 (12.3, 22.1)	<0.001
**L5**	16.9 ± 4.1 (10.2, 24.9)	14.9 ± 3.0 (9.9, 20.9)	19.3 ± 4.1 (11.9, 26.4)	<0.001
**S1**	15.1 ± 6.3 (6.3, 30.0)	11.9 ± 3.3 (6.0, 18.1)	19.1 ± 6.9 (7.6, 32.3)	<0.001
**Dural sac diameter ratio**				
**L1**	0.63 ± 0.12 (0.41, 0.87)	0.60 ± 0.11 (0.38, 0.79)	0.67 ± 0.12 (0.51, 0.92)	0.003
**L2**	0.57 ± 0.11	0.54 ± 0.09	0.60 ± 0.11	<0.001

	(0.36, 0.79)	(0.36, 0.72)	(0.40, 0.81)	
**L3**	0.52 ± 0.10 (0.35, 0.71)	0.49 ± 0.09 (0.33, 0.67)	0.55 ± 0.10 (0.37, 0.74)	0.002
**L4**	0.51 ± 0.12 (0.32, 0.75)	0.48 ± 0.10 (0.31, 0.69)	0.56 ± 0.11 (0.39, 0.78)	<0.001
**L5**	0.57 ± 0.16 (0.32, 0.90)	0.50 ± 0.12 (0.31, 0.74)	0.66 ± 0.16 (0.38, 0.98)	<0.001
**S1**	0.77 ± 0.55 (0.25, 2.33)	0.50 ± 0.14 (0.23, 0.79)	1.11 ± 0.68 (0.36, 2.74)	<0.001
**L5 nerve root sleeve diameter (mm)**	6.1 ± 1.1 (4.0, 8.1)	6.0 ± 1.0 (4.2, 8.0)	6.1 ± 1.2 (4.0, 8.5)	0.64
**S1 scalloping (mm)**	3.91 ± 2.15 (0.5, 9.1)	3.1 ± 1.3 (0.5, 5.8)	5.0 ± 2.5 (0.71, 9.8)	<0.001

Data are mean ± standard deviation (95% confidence interval). MFS = Marfan syndrome.
